# Supplementary material for: PCR diagnostics are insufficient for the detection of Diarrhoeagenic Escherichia coli in Ibadan, Nigeria
Source: PLOS Glob Public Health. 2023 Aug 7;3(8):e0001539. doi: 10.1371/journal.pgph.0001539 (PMC10406320; doi:10.1371/journal.pgph.0001539)
Supplement: Attachment — Submitted filename: Bisi DEC PCR and WGS reviewrs responsev3.docx [file pgph.0001539.s001.docx]

Response to review Manuscript ID- PGPH-D-22-02114

Editorial Requirements

0.1 The Editorial office requested for a reference list review to ensure that it is complete and correct. We have reviewed the reference list for accuracy.
0.2 The Editorial office requested for the removal of the copyright or trademark symbols we included with the original manuscript. We have removed the trademarks ® and ™ in the manuscript as verifiable on lines 133 and 142 of the revision.
0.3 The Editorial office requested that all figures should be in a separate files in .tif or .eps format only, that we remove any figures embedded in the manuscript file and ensure that all files are under the size limit of 10MB. We have removed all figures in the manuscript and saved them in tif format as recommended.
0.4 The Editorial office noticed that we have uploaded Supporting Information files, but did not include a list of legends. We did not in fact include supporting information files. All the data for the manuscript are included in the main manuscript or submitted to public repositories. The document uploaded as ‘supporting information’ on the journal system is a related but non-overlapping manuscript, which is currently under-review and also posted at MedRxiv.

Reviewer #1:

1.1. The reviewer mentioned that our article is well-planned and conducted. However, there are some points that need to be clarified before publication. We thank the reviewer for the positive comments and have addressed his/her concerns as outlined below.
1.2. The reviewer suggested that the title required rearranging. We have altered the original title from ‘Insufficiency of PCR diagnostics for Detection of Diarrhoeagenic Escherichia coli in Ibadan, Nigeria’ to read ‘Underperformance of Multiplex PCR for Detection of Diarrhoeagenic *Escherichia coli* Pathotypes in Ibadan, Nigeria’. We hope that the reviewer will find the revised title more suitable.
1.3. The reviewer asked that the conclusions be better aligned with the objectives. The objectives, as stated in lines 85-88 of the revised manuscript are to compare PCR with whole genome sequencing and to identify the root causes of PCR mis-identification. As the findings from the second aim were not well articulated in the conclusion, a suitable addition has been made in lines 430-433 of the revision.

Reviewer #2: 
2.1. The reviewer requested the details of the study population in the materials section. In response to the reviewer’s comment, this information has been added to lines 108-109 of the revised manuscript.

2.2. The reviewer requested for clarification about the number of specimens yielding *E. coli*. The epidemiology study enrolled 120 children with diarrhea and 357 healthy children, totaling 477 children. *E. coli* was then isolated from 413 specimens. In response to the reviewers’ comment, this is now reported on 113 of the revised manuscript.

2.3. The reviewer stated that some sentences in the Results section of the original manuscript, which explain the reason for the research findings, should be written in the discussion. We appreciate the reviewer for this observation. We have moved the sentence to the Discussion of the manuscript now in lines 342 to 345 of the marked-up copy of the revised manuscript.

2.4. The reviewer noted a typo in the discussion section, line 406 of the original manuscript. He requested we replace the “be” to “can”. On line 409 of the marked-up copy of the manuscript we have effected the requested change.
