# Supplementary material for: PCR diagnostics are insufficient for the detection of Diarrhoeagenic Escherichia coli in Ibadan, Nigeria
Source: PLOS Glob Public Health. 2023 Aug 7;3(8):e0001539. doi: 10.1371/journal.pgph.0001539 (PMC10406320; doi:10.1371/journal.pgph.0001539)
Supplement: Attachment — Submitted filename: PLoS GPH resp to review 2plus.docx [file pgph.0001539.s002.docx]

Response to editorial comments on ‘**PCR diagnostics are insufficient for the detection of Diarrhoeagenic *Escherichia coli* in Ibadan, Nigeria’**.

We thank you for the opportunity to fix issues with the manuscript.

1. The editor requested that we adjust our online Competing Interests statement. We have now added a statement to the effect that “The authors have declared that no competing interests exist.”
2. The editor observed that Figure 3 was not called out of the manuscript. Figure 3 is now called out from line 329 on the manuscript

Thank you and best wishes,

Olabisi C Akinlabi and Iruka N Okeke
